# Antimicrobial, anticancer, and biofilm inhibition studies of highly reduced graphene oxide (HRG): *In vitro* and *in silico* analysis

**DOI:** 10.3389/fbioe.2023.1149588

**Published:** 2023-03-16

**Authors:** Abdulaziz Alangari, Ayesha Mateen, Mohammed S. Alqahtani, Mudassar Shahid, Rabbani Syed, Mohammed Rafi Shaik, Mujeeb Khan, Syed Farooq Adil, Mufsir Kuniyil

**Affiliations:** ^1^ Department of Clinical Laboratory Sciences, College of Applied Medical Sciences, King Saud University, Riyadh, Saudi Arabia; ^2^ Department of Pharmaceutics, College of Pharmacy, King Saud University, Riyadh, Saudi Arabia; ^3^ Department of Chemistry, College of Science, King Saud University, Riyadh, Saudi Arabia

**Keywords:** highly reduced graphene oxide, antimicrobial, anticancer, *in vitro*, *insilco*

## Abstract

**Background**: Bacterial infections and cancers may cause various acute or chronic diseases, which have become serious global health issues. This requires suitable alternatives involving novel and efficient materials to replace ineffective existing therapies. In this regard, graphene composites are being continuously explored for a variety of purposes, including biomedical applications, due to their remarkable properties.

**Methods:** Herein, we explore, *in-vitro*, the different biological properties of highly reduced graphene oxide (HRG), including anti-cancer, anti-bacterial, and anti-biofilm properties. Furthermore, to analyze the interactions of graphene with proteins of microbes, *in silico* docking analysis was also carried out. To do this, HRG was prepared using graphene oxide as a precursor, which was further chemically reduced to obtain the final product. The as-prepared HRG was characterized using different types of microscopic and spectroscopic techniques.

**Results:** The HRG revealed significant cytotoxic ability, using a dose-dependent anti-cell proliferation approach, which substantially killed human breast cancer cells (MCF-7) with IC_50_ of 29.51 ± 2.68 μg/mL. The HRG demonstrated efficient biological properties, i.e., even at low concentrations, HRG exhibited efficient anti-microbial properties against a variety of microorganisms. Among the different strains, Gram-positive bacteria, such as B. *subtilis*, MRSA, and S. *aureus* are more sensitive to HRG compared to Gram-negative bacteria. The bactericidal properties of HRG are almost similar to a commercially available effective antibiotic (ampicillin). To evaluate the efficacy of HRG against bacterial biofilms*, Pseudomonas aeruginosa* and MRSA were applied, and the results were compared with gentamycin and ampicillin, which are commonly applied standard antibiotics. Notably, HRG demonstrated high inhibition (94.23%) against *P.aeruginosa*, with lower MIC (50 μg/mL) and IC_50_ (26.53 μg/mL) values, whereas ampicillin and gentamicin showed similar inhibition (90.45% and 91.31% respectively) but much higher MIC and IC_50_ values.

**Conclusion:** Therefore, these results reveal the excellent biopotential of HRG in different biomedical applications, including cancer therapy; antimicrobial activity, especially anti-biofilm activity; and other biomedicine-based therapies. Based on the molecular docking results of Binding energy, it is predicted that pelB protein and HRG would form the best stable docking complex, and high hydrogen and hydrophobic interactions between the pelB protein and HRG have been revealed. Therefore, we conclude that HRG could be used as an antibiofilm agent against P. *aeruginosa* infections.

## 1 Introduction

Recently, graphene and its derivatives, like highly reduced graphene oxide (HRG), have become popular materials for several technological applications (Khan et al., 2015). In particular, due to their exceptional biological properties, the use of these materials in the field of biomedicine has been increasing continuously over the last decade (Khan et al., 2016). Graphene is one of the most flexible and strongest substances and has a number of other intriguing qualities, including high stability, decent biocompatibility, and excellent electrical conductivity (Allen, Tung, and Kaner, 2010). Due to this, different types of graphene derivatives, like single-layer graphene Nanosheets, multi-layer graphene flakes, highly oxygenated graphene (graphene oxide, GO), highly reduced graphene oxide (HRG), etc. Have been extensively applied for different biomedical applications, including drug and gene delivery, bio-imaging, tissue engineering, and cancer therapy (Zhu et al., 2016; Karki et al., 2020). In general, the biopotential of graphene derivatives is mainly dependent on the size, shape, and contents of the resulting materials (Skoda et al., 2014).

For example, in the case of pristine graphene, which only consists of a carbon network, its biological potential is highly inhibited by its low dispensability in different solvents (Yang et al., 2013). On the other hand, HRG exhibits decent dispensability in aqueous and other solutions due to the existence of oxygenated functional groups on its surface, which lead to the enhancement of the solubility of the resulting material (Jaworski et al., 2021). This results in increased acceptability of HRG for a variety of bio-medical and bio-medicinal studies. For example, HRG delivers excellent cytotoxic effects depending on the size, surface charge, and nature of oxygenated groups, which heavily contribute to the toxicity of the material (Sengupta et al., 2019). Therefore, in comparison to pristine graphene, HRG is more likely to produce low toxicity and decent biological potential (Jagiełło et al., 2020). In addition, the preparation of pristine graphene on a large scale for biological applications is more challenging due to graphite’s unique use as a precursor of graphene (Compton and Nguyen, 2010). However, the preparation of HRG is easier *via* chemical exfoliation approaches involving successive oxidation and the reduction of graphite, which leads to the formation of heavily oxygenated and charged graphene-like nanosheets (HRG) (Adil et al., 2022).

To date, various studies have explored the influence of graphene and its derivatives on a variety of microbes such as fungi, bacteria, cancerous cells, etc. (Wojtoniszak et al., 2012). However, most of the existing studies have inconsistent and inconclusive results, which is possibly attributed to different experimental conditions, types of graphene-based materials, and their preparation methods (Kavinkumar et al., 2017a). Thus, to utilize the full potential of graphene derivatives, different types of graphene derivatives with diverse functionalities have been extensively investigated for various biomedical applications, including treating bacterial infections and different types of cancers (Shafiee, Iravani, and Varma, 2022). In particular, in the case of anti-cancer activities, such as tumor therapy, graphene derivatives have received great interest, as they have so far generated diverse effects on both cancerous and normal cells (Rahimi et al., 2022). Furthermore, graphene-based materials are mainly comprised of carbon, which is generally considered a safe element for humans and other living organisms, and these materials have demonstrated ultimate biocompatibility (Pinto, Goncalves, and Magalhaes, 2013). Due to their small size and sharp edges, these materials have also been known for their easy penetration of cells, which is a prerequisite for diagnosis and other applications (Su et al., 2017).

Besides cancer, another leading cause of illness, physical impairments, and mortality worldwide is bacterial infections, which are mostly related to 33 types of bacterial species (Ikuta et al., 2022). Fortunately, antibiotics and other effective drugs have long protected humans from deadly bacterial infections (Paterson, 2019). However, extensive use of conventional antibiotics has facilitated the undesired evolution of a variety of drug-resistant bacteria, which are currently responsible for several lethal infections (Opal, 2016). In most cases of multi-drug resistant (MDR) infections, traditional antibacterial materials like regular antibiotics, herbal products, metal ions/oxides, quaternary ammonium compounds, and so on, have been less effective and have presented various diseases (Khorsandi et al., 2021; Huang et al., 2022). Therefore, due to the large-scale prevalence of MDR infections, scientists and medical experts have considered various non-conventional anti-bacterial substances, such as metallic and metal oxide nanoparticles, carbonaceous materials including graphene derivatives, etc., (MakabentaJessa Marie et al., 2021). Of these materials, graphene derivatives have so far demonstrated remarkable characteristics in combating different types of bacterial infections (Szunerits and Boukherroub, 2016). The toxicity of graphene derivatives against bacteria can be attributed to both physical damage and chemical interactions (Ji, Sun, and Qu, 2016). The physical mode of action involves the direct interactions of the sharp edges of graphene with the bacterial cell wall and/or photothermal ablation and wrapping of bacterial species, which ultimately damages them (Feng et al., 2019). The chemical toxicity in graphene is possibly caused by oxidative stress, which occurs due to the presence of reactive oxygen species (ROS) and charge transfer, which are largely present in graphene derivatives (Yaragalla, Bhavitha, and Athanassiou, 2021).

Furthermore, due to their small size, high specific surface area, and other unique physicochemical properties, graphene derivatives are also effective in inhibiting the formation of bacterial biofilm (Cao et al., 2021). Graphene-based drug delivery Nanocarriers have been known to facilitate the controlled release of antimicrobials into biofilm-infected tissues to enhance the availability and decrease the adverse side effects of antibiotics (Liu et al., 2021). In addition, photothermal graphene-based materials locally generate heat under the influence of light, which leads to the thermal ablation of bacteria for the photothermal therapy (PTT) of biofilm infections (Wang et al., 2020). Despite the fact that studies investigating the interactions between graphene derivatives with various mammalian cells to explore the regulating factors of their *in vitro* and *in vivo* toxicity are increasing at a great pace (Zuchowska et al., 2017), many of these studies are mainly focused on investigating the effect of graphene derivatives on selected cell cultures.

Thus, for a detailed exploration of the interactions of graphene derivatives with microbial and other biological entities, more comprehensive and diverse studies on different microorganisms are required. Therefore, herein, to explore the biological potential of graphene, we synthesized and oxygenated a derivative of graphene, which is referred to as highly reduced graphene oxide (HRG). The as-prepared HRG was characterized using a variety of techniques including UV, FT-IR, XRD, Raman, and TEM. The HRG is further used to investigate the antibacterial and anti-biofilm potential against a variety of bacterial strains. It has also been applied to evaluate the cytotoxic effect on MCF-7 human epithelial cells for therapeutic application in the treatment of breast cancer. All the biological experiments were performed *in vitro*, but to understand the interactions of HRG with the biological entities (proteins), *in silico* molecular docking analysis was performed.

## 2 Materials and methods

### 2.1 Materials

N2H4 (50–60%), KMnO4 (99%), H2O2 (30 wt%), H2SO4 (98%), and NaNO3 (99%) were procured from Sigma-Aldrich and used as such. Graphite powder (99.999%, −200 mesh) was delivered from Alfa Aesar.

### 2.2 Preparation of graphite oxide (GRO)

The precursor of HRG, i.e., graphene oxide (GO) was prepared according to our previously published study, which followed a modified version of Hummers’ method (Al-Marri et al., 2015). Details of the preparation are presented in the supplementary information.

### 2.3 Preparation of highly reduced graphene oxide (HRG)

In order to prepare the highly reduced graphene oxide (HRG), freshly prepared graphene oxide suspension is transferred into a 100 mL round bottom flask, which is fitted with a cooling condenser. The suspension was allowed to heat up to 100°C, and subsequently, 3 mL of hydrazine hydrate was poured with continuous stirring. Thereafter, the temperature of the reaction was slightly reduced to 98 °C and the stirring was continued for 24 h. After this, the suspension was filtered, and the solid black residue was washed several times with DI water. The product was collected *via* centrifuge at 4,000 rpm and dried in a vacuum.

### 2.4 Characterization techniques

The details of the instruments used for the characterization of the samples are presented in the supplementary information.

### 2.5 Cytotoxicity analysis

The cell antiproliferative analysis of HRG was conducted using MCF-7 human epithelial cells, which were obtained from the department of pharmaceutics, King Saud University. The cells were then grown in DMEM (Gibco, UK), including 1% penicillin and streptomycin in an incubator of 5% CO2 at 37°C, with 10% fetal bovine serum (Gibco, UK) and 1% antibiotics. MCF-7 cells were exposed to HRG at different concentrations, ranging from 1.56 to 200 μg/mL. After incubation for 24–48 h, the cell suspension was washed with PBS buffer. MTT (2,5-Diphenyl-2H-Tetrazolium Bromide) standard solution with a concentration of 5 mg/mL was prepared, 20 µL of the MTT solution was added to the wells, and the plates were incubated for 4 h at 37 °C. The MTT containing culture fluid was then removed, leaving the formazan crystal to precipitate. For 15 min, the crystals were dissolved in 100 µL of DMSO/acetic acid/sodium lauryl sulfate (99.4 mL/0.6 mL/10 g). The absorbance at 570 nm was calculated using a spectrophotometric microplate reader (Synergy HT, BioTek Inst., Winooski, VT, USA). Graph Pad Prism 5.0 was used to compute the IC50 (San Diego, CA 92108, USA).

### 2.6 Screening of synthesized HRD for antimicrobial analysis

Antimicrobial assessment of HRG using the agar diffusion method was conducted using four pathogenic bacterial strain names. E. *coli*, P. *auroginosa*, B. *subtilis*, and Methicillin-resistant Staphylococcus *aureus* (MRSA) were collected from the Pharmaceutics Microbiology department, King Saud University. In short, bacterial cultures were subcultured and fresh cultures were prepared in Muller Hilton agar broth, and 0.5 McFarland stranded culture of each test culture was plated on MHA agar plates. Stock concentrations of HRG nanoparticles and antibiotic standard drug ampicillin were prepared, and 100 µL of both HRG and ampicillin were poured into wells that had been prepared on agar plates. All the test plates were prepared in triplicate and incubated for 24 h at 37°C. After incubation, the zone of inhibition (ZOI) diameter was measured with a scale.

### 2.7 Determination of minimum biofilm inhibitory concentration (MBIC)

To evaluate the HRG nanoparticles’ antibiofilm activity, a static microtiter plate assay was performed. Inoculums of 100 μL of P. *aeruginosa* and MRSA strains were grown in polystyrene, flat-bottom 12-well microplates for 24 h at 37°C (Corning, NY, United States). After the supernatant was removed following incubation, the wells were then cleaned twice with normal saline sterile. Standard antibiotics, including ampicillin 1000 μg/mL and gentamycin 200 μg/mL, were added to the wells along with 100 μL of nanoparticle stock (1 mg/mL) and antibiotics to achieve concentrations of 6.25–1000 micrograms with the formed biofilms. After being cultured for 18 h at 37°C, the cells were examined using an inverted microscope (Olympus, Tokyo, Japan) set at ×40 magnification, MBIC was then recorded as the lowest concentration of nanoparticle that produced no visible growth.

### 2.8 Docking studies

The 2D structure of reduced graphene was sketched using the ACD/ChemSketch software and Avogadro software was used to optimize Geometry and generate a PDB file of ligand HRG. Further, the RESP charge Calculations were done using the RED Server (https://upjv.q4md-forcefieldtools.org/REDServer-Development).

The protein sequence and the PDB file of PelB protein from P. *aeruginosa* PAO1 were obtained from the RCSB website (https://www.rcsb.org/structure/5WFT).

>5WFT_1|Chain A|PelB|*Pseudomonas aeruginosa* (strain ATCC 15692/DSM 22644/CIP 104116/JCM 14847/LMG 12228/1C/PRS 101/PAO1) (208,964).

EDRTLLADLARLGEWTGNGPRALGFWKQLLAGADDPALREHAWRLSLQMFDFDSAIELLAPIGAQRQMTDEELDALVYSHETRGTPEEGEAWLRGYVQRYPKQRLAWQRLQQILEHTQ

We used the AutoDock 4.2 program, which uses auto dock tools to assign polar hydrogens, unified atom Kollman charges, solvation parameters, and fragmental volumes to the protein. Molecular docking procedures are frequently used to predict the binding affinities of a variety of ligands. The prepared file was saved by Auto Dock in PDBQT format. A grid map was created using Auto Grid and a grid box. A scoring grid was created using the ligand structure to speed up computation time. The grid center was set to 1.095, 1.554, and 3.894 in the x, y, and *z*-axes, with a grid size of 60 60 60 xyz points and a grid spacing of 0.375 A. PyMOL was used to visualize the resulting docked complex, and a subsequent docking analysis was carried out using the protein-ligand interactions (https://plip-tool.biotec.tu-dresden.de/plip-web/plip/index) (Adasme et al., 2021).

### 2.9 Statistical analyses

The statistical analysis for cytotoxicity and the antimicrobial assessment of HRG were analyzed using Prism software, and a *p*-value of 0.05 was considered statistically significant.

## 3 Results and discussion

### 3.1 UV-Vis analysis

**FIGURE 1:**
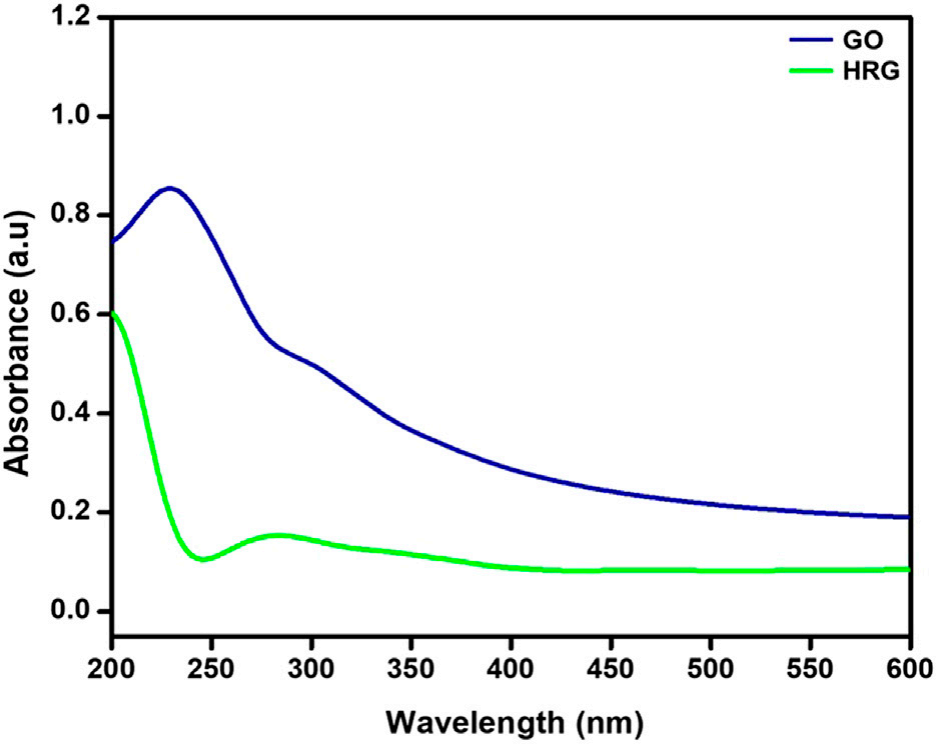
UV-Vis absorption spectra of Graphite oxide (GO) and highly reduced graphene oxide (HRG).

The initial confirmation of the preparation of HRG was carried out by UV analysis. Typically, GO exhibits a broad peak between 200 and 250 nm, which shifts to a higher wavelength upon reduction. During the reduction process, the majority of oxygenated groups from the surface of graphene oxide Nanosheets are depart, leading to the restoration of aromatic conjugation (Vinoth et al., 2015). Similarly, in the case of HRG prepared in this study, the peak of GO appears at ∼230 nm (blue line, Figure 1), which can be attributed to the *π*-π* transition of the C=C bond of the aromatic ring and n-π* transition of C=O bonds. However, upon reduction, the peak shifts to the higher wavelength and relocates at ∼283 nm (green line, Figure 1), possibly indicating the reduction of GO (Paredes, 2008).

### 3.2 FT-IR analysis

**FIGURE 2:**
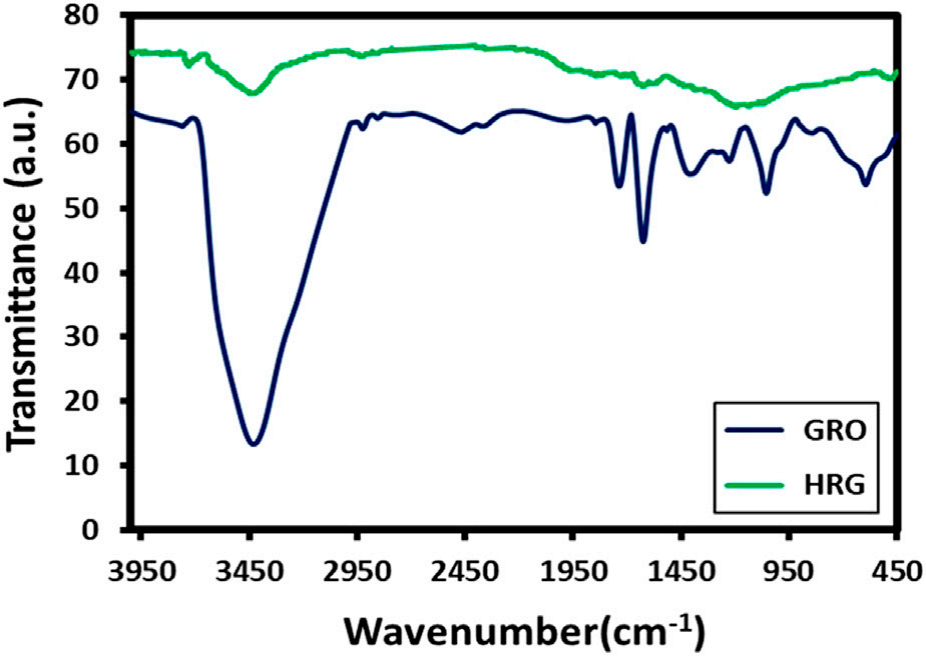
FT-IR spectra of Graphite oxide (GRO) and highly reduced graphene oxide (HRG).

Due to the presence of a large number of oxygenated groups on the surface of GO, FT-IR is a suitable technique for analyzing diverse functional groups of both GO and HRG. To perform this, FT-IR spectra of both GO and HRG were measured and plotted in Figure 2. The oxygenated groups of GO generate plenty of IR signals; the region between 1000 and 1800 cm-1, in particular, contains a large number of peaks. The oxygenated groups of GO are comprised of diverse functional groups involving carbon and oxygen, which include carbonyl (C=O), etheric (C-O-C), and alcoholic (C-O) functionalities. These groups generate IR signals at various frequencies, such as 1735 cm-1 (stretching), 1400 cm-1 (bending), 1224 cm-1 (stretching), 1053 cm-1 (stretching), and so on (RagupathyNarayanan and Pattanayak, 2014). Apart from functional groups involving carbon and oxygen, the IR signals are also generated from other functionalities involving hydroxyl groups (OH), which possibly appear as a broad peak between 3,200 and 3,500 cm-1; in this case, it appears at 3,428 cm-1 in the FTIR spectrum of GO (blue line, Figure 2). Usually, after the reduction of GO, the majority of these functional groups disappear, but some of them remain as it is not possible to completely remove these functional groups due to experimental constraints. Due to this, the IR signals present in GO may not completely disappear but may be present with significantly reduced intensities (Trivedi et al., 2015). As expected, the IR spectrum of HRG (green line, Figure 2) exhibits similar IR peaks to that of GO, but their intensities are considerably reduced, indicating the reduction of GO. In FT-IR spectra of HRG (Figure 2), the exclusion of such oxygen comprising groups of GO in HRG was specified by the disappearance of some of the bands in their respective FT-IR spectra, such as the bands at ∼1735 and ∼1630 cm−1. Also, the comparative intensity decrease in some of the other bands, like the decrease in intensity of the broad band at 3,440 cm−1 associated with the hydroxyl groups of GO, points in the direction of GO reduction.

### 3.3 XRD analysis

**FIGURE 3:**
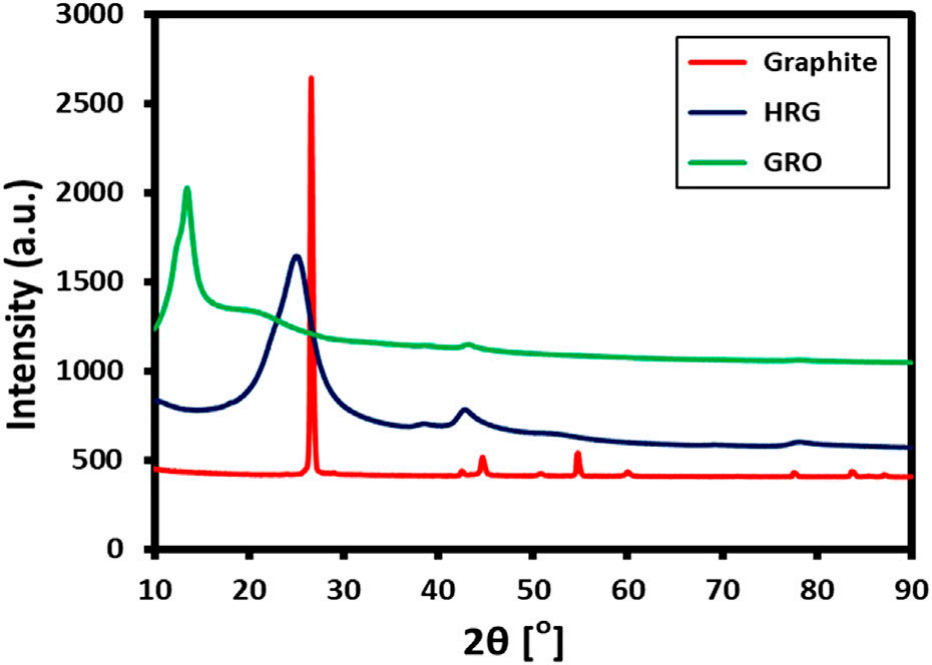
XRD diffractograms of Graphite oxide (GRO) and highly reduced graphene oxide (HRG).

XRD analysis was performed to check the crystallinity of as-prepared HRG, and its XRD pattern was compared with other precursors, such as GO and pristine graphite. Figure 3 displays the XRD pattern of HRG (blue line), GO (green line), and graphite (red line). Since pristine graphite is highly crystalline, it exhibits a sharp characteristic peak at 26.5° (002), with a d-spacing of 0.34 nm, which is calculated using Bragg’s equation (Divya et al., 2018). However, this changes drastically after the oxidation process, which induces severe defects in the crystalline network of graphite. Due to this, the sharp peak of graphite at 26.5° shifts to 13.4° (001) and appears as a broad reflection with increased interplanar distance (0.66 nm). Additionally, the XRD spectrum of GO also displays a small shoulder peak at 42.8°, which corresponds to (004) or (100) planes (Hassan et al., 2013). However, in the case of HRG, most of the functional groups are departed from GO, and the graphitic structure is partially restored; thus, the XRD reflection again shifts toward a higher angle and appears at 23.4° (002) in their diffraction patterns indicating the formation of graphene nanosheets with a thickness of few layers. Notably, the XRD reflection of HRG is considerably broad when compared to the XRD peak of pristine graphite. This indicates the relatively low crystallinity of HRG compared to its precursor, graphite. In addition, the decreasing interplanar distance observed in HRG, with respect to GO, indicates the formation of HRG. The sp2 hybridization of the graphitic carbon is retained in HRG. As observed in Figure 3, the diffraction peaks corresponding to graphite (2θ = 26.5°) and GO (2θ = 13.4°) are completely absent in the XRD pattern of HRG, which clearly points toward the formation of a crystalline intermediate, i.e., HRG.

### 3.4 Raman analysis

**FIGURE 4:**
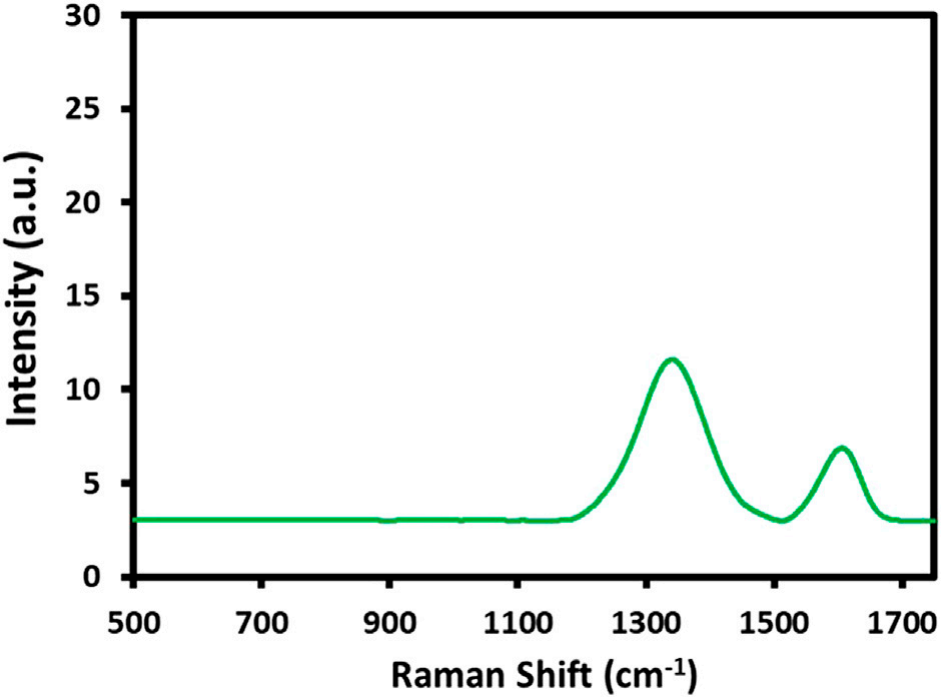
Raman analysis of highly reduced graphene oxide (HRG).

In addition, the Raman analysis of HRG was also performed, which is an efficient technique for obtaining information about the functional fragments that may appear during the chemical transformation of graphite to GO and HRG (Kudin et al., 2008). The Raman spectrum of HRG in Figure 4 shows two characteristic bands at 1595 and 1360 cm-1, which correspond to the D band and G bands, respectively. In the case of pristine graphene, the in-phase vibration of the graphite lattice (G band) usually appears at 1575 cm-1, and the disorder band caused by the graphite edges (D band) occurs at 1355 cm-1 (data not shown here). On the other hand, the G and the D bands of GO slightly shift to a higher frequency and appear at 1592 and 1346 cm-1 (data not shown here) (Khan et al., 2014). Notably, the peak locations of the G and D bands of HRG obtained in this study do not match both the reported values of pristine graphite and GO, which may indicate the formation of HRG.

### 3.5 Transmission electron microscope (TEM) analysis

**FIGURE 5:**
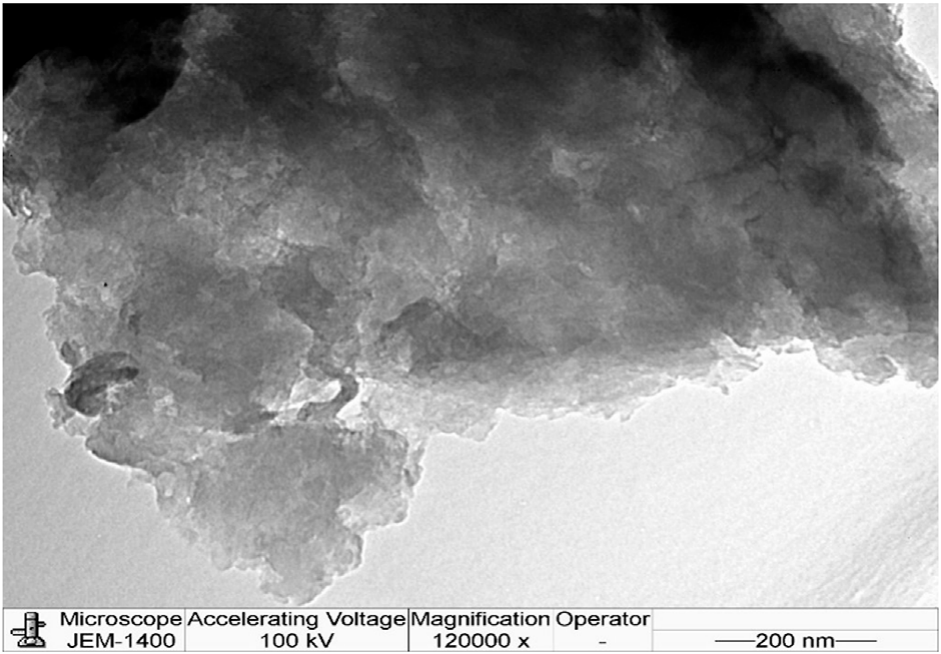
HRTEM analysis of highly reduced graphene oxide (HRG).

The HRTEM analysis of HRG is shown in Figure 5 and displays the structure and layer thickness of the HRG. The obtained data show that sheets consist of a few layers stacked on top of each other, with some wrinkles and foldings. A large number of wrinkles and scrolls were noticed on the HRG surface, which constantly endured the high-energy electron beam. Figure 5 signifies an HRTEM micrograph of HRG sheets and displays the graphene lattice fringes. This provides further information about the interplanar distance of HRG material.

### 3.6 Scanning electron microscope (SEM) analysis

**FIGURE 6:**
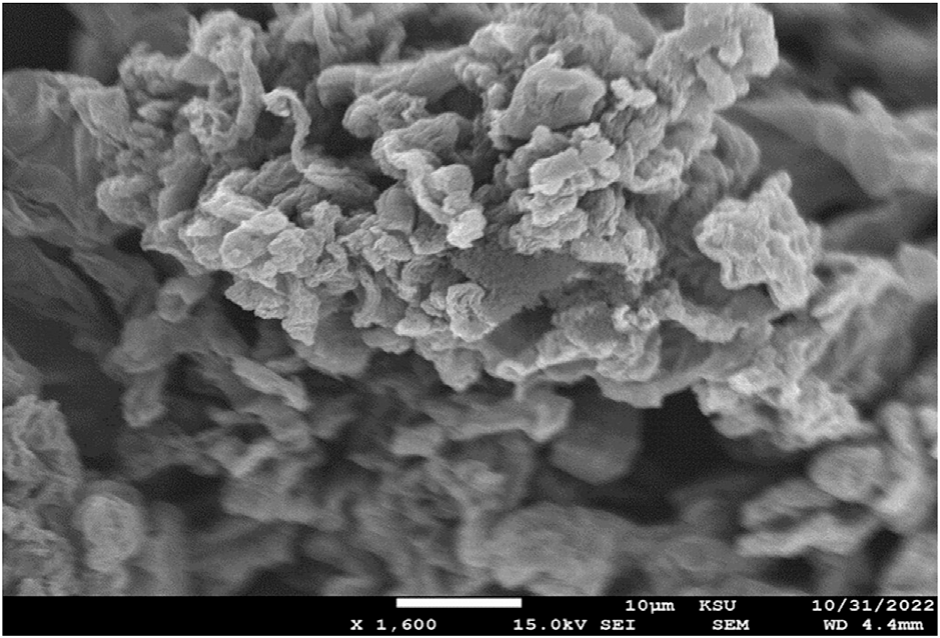
SEM analysis of highly reduced graphene oxide (HRG).

The reduction of graphite oxide may induce some morphological changes from the original structure of pristine graphite and GO; these changes can be effectively observed using scanning electron microscopy. During the HRG synthesis using an oxidation-reduction approach, the layers in the graphite were exfoliated. Due to the reduction, the attained HRG has a completely different morphology. The HRG exhibited in Figure 6 has a porous structure. The SEM image in Figure 6 displays the surface morphology of HRG, which was observed as similar thin sheets aggregated randomly, with different edges, wrinkles, and scrolled surfaces.

### 3.7 Cell proliferation assay

**FIGURE 7:**
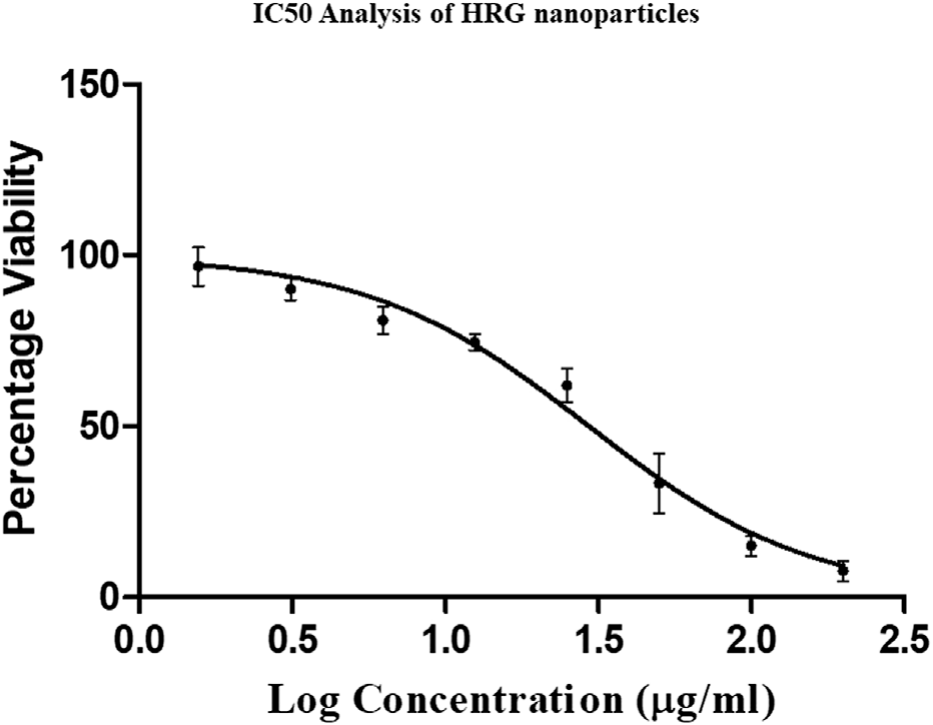
MTT assay showing IC 50 value of 29 μg/mL with MCF-7 cell lines incubated for 24 h.

The current trends in diagnostics and therapeutics in the treatment of cancer are mainly based on nanobiotechnology, which is attracting global attention in approaching individualized treatment (Chaturvedi et al., 2019). The results of the MTT (4,5-dimethylthiazol-2-yl)-2,5-diphenyltetrazolium bromide) assay show that HRG has a dose-dependent anti-cell proliferation effect on MCF-7. Figure 7 illustrates the percentage cell viability of MCF-7 breast cancer cells in response to various HRG doses. At 1.56–200 μg/mL, HRG was tested for cell proliferation, and the measured IC50 for MCF-7 was 29.51 ± 2.68 μg/mL at 24 h. A recent study produced updated results showing that reduced graphene oxide nanoparticles are cytotoxic toward tested human breast MCF cell lines (Smina et al., 2021). Precision medicine has profited from the successful use of nanotechnology to create novel therapeutic delivery methods using nanoparticles (NPs). Advances in nanoparticle engineering have enabled the use of NPs to substantially improve efficacy while addressing heterogeneous delivery hurdles (Mitchell et al., 2021). A recent research study concluded that reduced graphene significant cytotoxicity with IC50 30 μg/mL in tested cell lines when compared to graphene oxide (Varunkumar et al., 2017b).

### 3.8 Antibacterial analysis of HRG

We adopted an agar diffusion assay for the assessment of susceptibility to tested pathogenic strains. Our findings are summarized in Table 1. We selected both Gram-positive and Gram-negative pathogenic strains in this study. Gram-positive bacteria such as B. *subtilis*, MRSA, and S. *aureus* exhibited susceptibility to HRG nanoparticles when compared to Gram-negative bacterial strains. Gram-negative E. *coli* exhibited resistance to HRG with a zone of inhibition of (21.45 ± 1.52) in comparison to standard ampicillin with a zone of inhibition of (19.16 ± 1.72) (*p* ≥ 0.05). Gram-negative P. *aeruginosa s*howed susceptibility to HRG with a zone of inhibition of (27.1 ± 1.17) in comparison to ampicillin, with a zone of inhibition of (20.5 ± 1.75). Gram-positive strains MRSA, S. *aureus*, and B. *subtilis* showed susceptibility to HRG with a zone of inhibition of (28.7 ± 2.21, 24.31 ± 1.98 & 24.12 ± 1.17) in comparison to ampicillin with a zone of inhibition of (21.0 ± 2.27, 19.21 ± 1.82 & 18.86 ± 1.21), respectively (*p* ≤ 0.05). A previous study found that *E. coli* showed resistance to reduced graph oxide nanoparticles, although it was susceptible to a high concentration of these nanoparticles (Mann et al., 2021). Graphene and its derivatives also show a valuable impact in tissue engineering and exhibit strict antimicrobial activities. Graphene and its derivatives are suitable candidates for creating Nano hybrid structures, which are useful in various biomedical fields like tissue differentiation, regeneration, and infection control (Shang et al., 2019). As a result of the indiscriminate use of antibiotics, numerous drug-resistant bacteria have emerged, necessitating the search for new antimicrobial medicines. Several unconventional materials, such as metallic and metal oxide nanoparticles, as well as carbon-based compounds, such as nanotubes and graphene, have been studied (Faiz et al., 2018; Gunawan et al., 2020). The expected mechanism of antimicrobial action of HDR may be due to both membrane and oxidation stress, as confirmed by previous studies.

**TABLE 1:** ZOI results from agar diffusion test by HRG, as compared to ampicillin. Results were presented as mean ± SD, n = 3.

Microorganisms	Zone of inhibitions (mm), mean ± SD, n = 3	
	Ampicillin	HRG
P. *aeruginosa*	20.5 ± 1.75	27.1 ± 1.17**
MRSA	21.0 ± 2.27	28.7 ± 2.21 **
S*. aureus*	19.21 ± 1.82	24.31 ± 1.98 *
E. *coli*	19.16 ± 1.72	21.45 ± 1.52
B. *subtilis*	18.86 ± 1.21	24.12 ± 1.17 *

### 3.9 Biofilm inhibition assay

**FIGURE 8:**
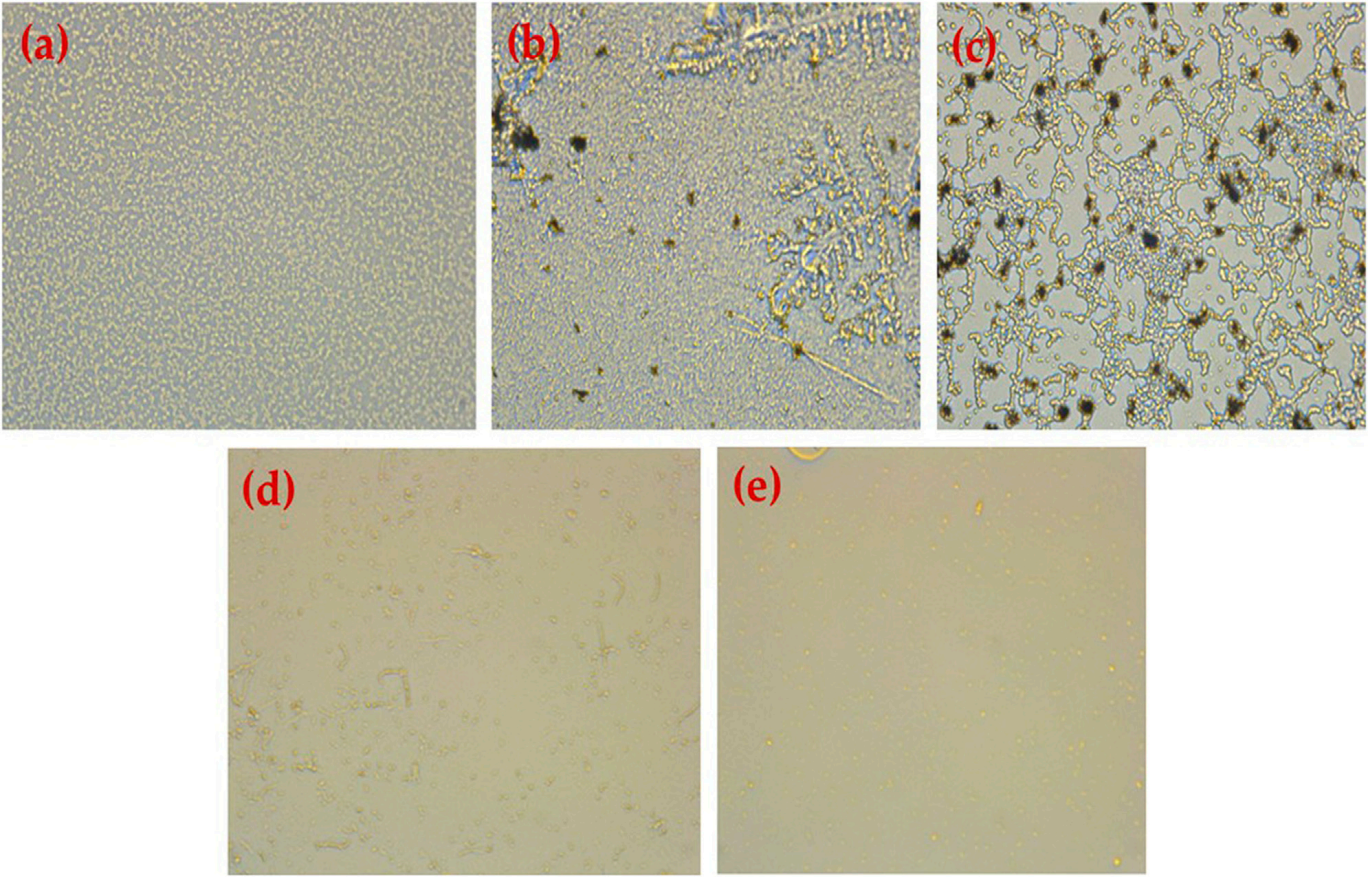
Results obtained using an inverted microscope (40) **(A)** untreated *P. aeruginosa* biofilm, **(B)** *P. aeruginosa* treated with HRG 100 (µg/mL), **(C)** *P. aeruginosa* treated with HRG 200 (µg/mL), **(D)** *P. aeruginosa* treated with Ampicillin 1 mg/mL, and **(E)** *P. aeruginos* treated with Gentamycin (100 mg/mL).

**FIGURE 9:**
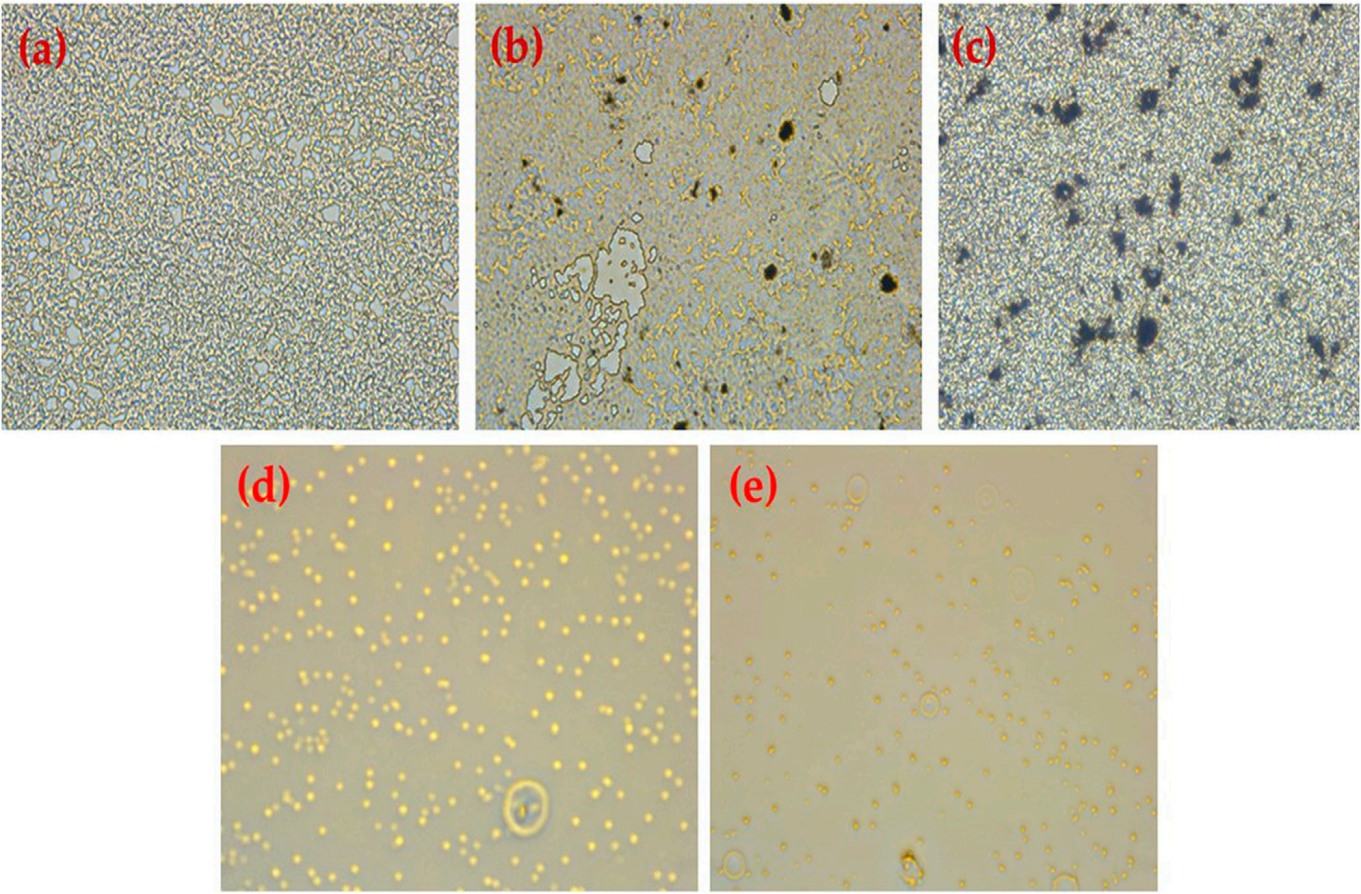
Results obtained using an inverted microscope (40) **(A)** untreated MRSA biofilm, **(B)** MRSA treated with HRG 100 (µg/mL), **(C)** MRSA treated with HRG 200 (µg/mL), **(D)** MRSA treated with Gentamicin 100 (µg/mL), and **(E)** MRSA treated with ampicillin (1 mg/mL).

The anti-biofilm activity of HRG was estimated using two pathogenic strains: P.*aeruginosa* and MRSA, which are related to the standard antibiotics gentamicin and ampicillin, and the results are shown in Table 2. Good biofilm formation was observed to be treated with HRG, and the results were in agreement with previous studies where reduced graphene showed antibiofilm activity in tested isolates. Biofilm inhibition action of HRG against P. *aeruginosa* showed the highest inhibition of 94.23%, with a MIC of 50 μg/mL and an IC50 of 26.53 μg/mL, whereas ampicillin and gentamicin showed inhibition of 90.45% and 91.31%, with a MIC of 200 μg/mL and 1000 μg/mL, respectively, and the inverted microscopic results are shown in Figure 8. The MRSA biofilm inhibition activity with HRG showed 93.76% inhibition with a MIC value of 100 μg/mL and a relative IC_50_ of 53.32 μg/mL. Both ampicillin and gentamicin inhibit MRSA biofilm with higher concentrations of 200 μg/mL and 250 μg/mL in comparison to HRG, and concentration inverted microscopic results are included in Figure 9. Graphene and materials made from it (GMs) showed a variety of antibacterial activities against viruses, fungi, and bacteria (Alangari et al., 2022). The primary source of these effects is thought to be the direct physicochemical contact between GMs and bacteria, resulting in the fatal destruction of biological components, primarily proteins, lipids, and nucleic acids (Mohammed et al., 2020).

**TABLE 2:** Biofilm Evaluation of MIC, Percentage inhibition, and IC50.

S.No	Organisms	Drug	MIC (µg/mL)	Parentage inhibition	IC_50_ (µg/mL)
1	***P. aeruginosa***	HRG	100	94.23	26.53
		Gentamicin	200	90.45	110.55
		Ampicillin	1000	91.31	547.58
2	**MRSA**	HRG	100	93.76	53.32
		Gentamicin	200	89.79	111.37
		Ampicillin	250	91.07	137.25

### 3.10 Molecular docking analysis

**FIGURE 10:**
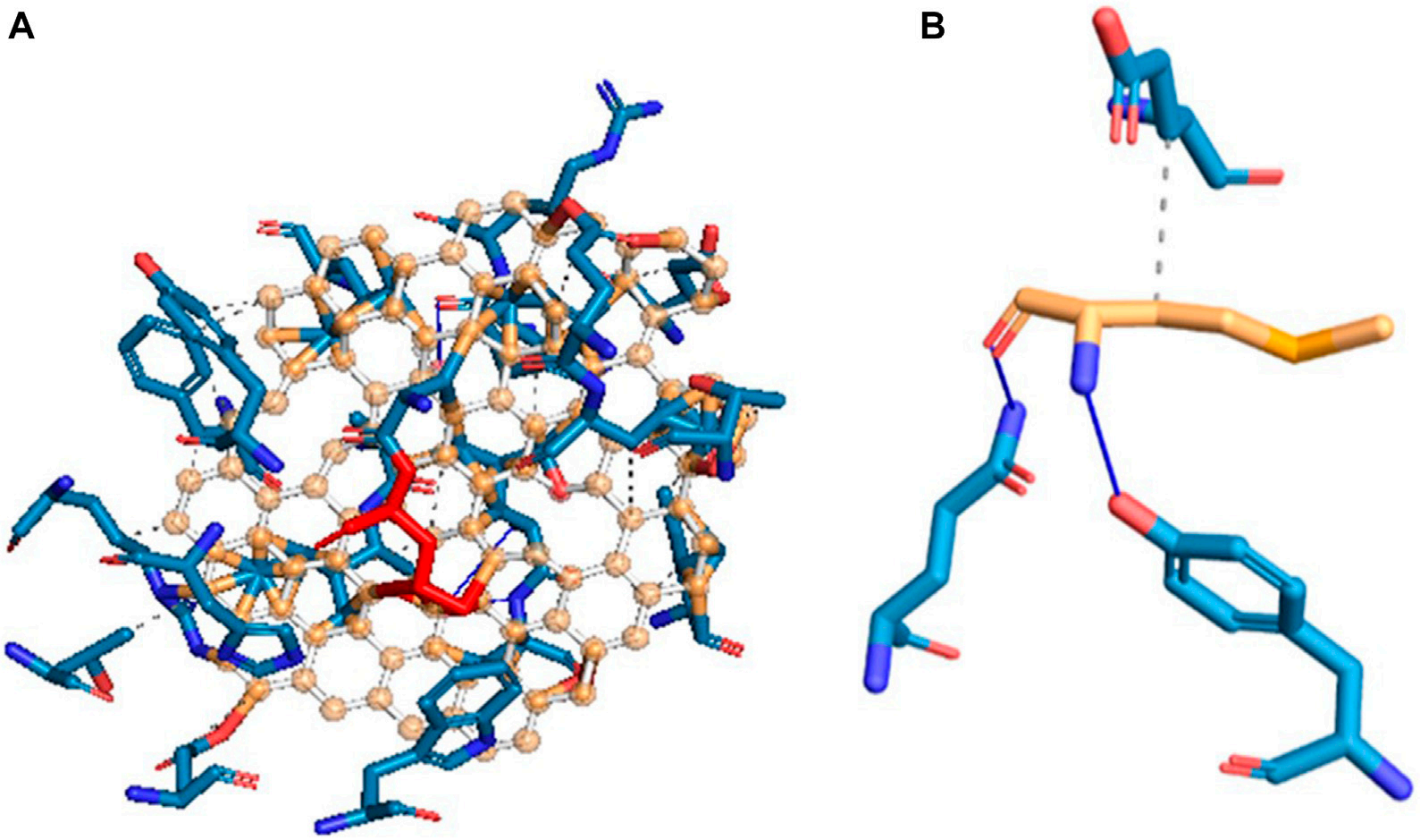
Hydrogen bond and hydrophobic interactions of **(A)** pelB protein and **(B)** HRG.

Molecular docking is a powerful computational approach to investigating ligand binding to the protein molecule at the atomic level. In the present *in silico* studies, the PelB protein from P. *aeruginosa* (Marmont et al., 2017) was docked with the ligand-reduced graphene using AutoDock 4.2 software, and the docking results showed docking binding-free energy of 4.31 kcal/mol and an RMSD value of 115.702 A in the high cluster docking (Table 3). Furthermore, the analysis was performed using PLIP software to analyze atomic levels of pelB protein and HRG interactions (Table 4 and 5; Figure 10).

**TABLE 3:** Binding energy and reference RMSD values of PelB and HRG complex.

Receptor	Ligand binding energy	Reference RMSD	
PelB Protein	Reduced Graphene	4.31 kcal/mol	115.702 A

**TABLE 4:** Hydrogen Bonds angle between docking complex atoms Index Residue AA Distance H-A Distance D-A Donor Angle Donor Atom Acceptor Atom.

Index	Residue (A)	AA	Distance H-A		Distance D-A	Donor angle	Donor atom	Acceptor atom
1	361	TRP	2.74		3.45	130.12	1051 [O3]	319 [O2]
2	377	LEU	3.55		3.98	108.81	446 [Nam]	1049 [O3]
3	378	ALA	1.83		2.27	102.97	454 [Nam]	1049 [O3]

**TABLE 5:** Hydrophobic Interactions between ligand and receptor atoms.

Index	Residue (A)	AA	Distance	Ligand atom	Protein atom
1	358	GLU	3.1	1037	297
2	361	TRP	1.54	1035	329
3	361	TRP	1.66	1015	327
4	361	TRP	1.66	1032	328
5	361	TRP	3.38	1012	323
6	361	TRP	3.05	1014	326
7	362	ARG	3.63	1040	335
8	365	LEU	1.33	1029	361
9	365	LEU	1.02	1025	361
10	365	LEU	2.13	1031	359
11	365	LEU	1.01	1009	362
12	370	PHE	2.99	1023	399
13	370	PHE	2.33	995	397
14	370	PHE	2.76	981	395
15	373	ALA	2.29	993	420
16	374	ILE	3.99	984	425
17	374	ILE	1.11	956	427
18	374	ILE	0.97	965	426
19	374	ILE	1.39	950	428
20	377	LEU	1.19	1012	453
21	377	LEU	0.72	999	452
22	377	LEU	1.58	987	450
23	378	ALA	1.29	958	458
24	380	ILE	3.11	1019	473
25	380	ILE	1.4	1004	472
26	380	ILE	2.11	991	470
27	387	THR	2.99	1047	514
28	389	GLU	3.28	1034	528
29	390	GLU	1.16	1027	537
30	390	GLU	3.87	1001	536
31	393	ALA	1.95	1010	561
32	394	LEU	3.43	999	566
33	394	LEU	1.06	974	569
34	394	LEU	1.72	969	568
35	396	TYR	3.3	1023	586
36	396	TYR	3.51	994	584
37	398	HIS	3.76	967	599
38	401	ARG	2.16	980	626
39	403	THR	3.04	967	642
40	406	GLU	3.52	949	664
41	410	TRP	3.57	973	697

## Data Availability

The original contributions presented in the study are included in the article/Supplementary Material, further inquiries can be directed to the corresponding author.
