# Supplementary material for: Antimicrobial, anticancer, and biofilm inhibition studies of highly reduced graphene oxide (HRG): In vitro and in silico analysis
Source: Front Bioeng Biotechnol. 2023 Mar 16;11:1149588. doi: 10.3389/fbioe.2023.1149588 (PMC10071309; doi:10.3389/fbioe.2023.1149588)
Supplement: Supplementary file 1 [file DataSheet1.docx]

**Preparation of Graphite oxide** **(GRO)**

The precursor of HRG i.e., graphene oxide (GO) is prepared according to our previously published study which followed a modified version of Hummers method. Initially, 2 g of natural graphite and 1.75 g of NaNO_3_ (purity 99%) were taken in a three-neck flask, to which 150 mL of H_2_SO_4_ (98%) was slowly added. The mixture was allowed to stir for 2 h under ice-water, after 2 h, 9 g of KMnO_4_ (99%) were slowly added under constant stirring over a period of 2 h. The remaining mixture was then allowed to react for five days at room temperature. Thereafter, 200 mL of 5 wt % H_2_SO_4_ aqueous solution were added over a period of 1 h, and the solutions was stirred for 2 h. Subsequently, 6 g of 30 wt % H_2_O_2_ aqueous solution were added, and the mixture was left for stirring for another 2 h. The resulting solution was thoroughly washed with an aqueous solution containing 3 wt % H_2_SO_4_ and 0.5 wt % H_2_O_2_ several times and finally three times with deionized water (DI). The resultant mixture was dispersed in DI water and centrifuged for 2 h at 9000 rpm. The resulting dispersion was purified by washing with DI water 20 times to obtain a brown-black homogeneous dispersion.

**Characterization techniques**

The as-synthesized HRG nanoparticles were characterized by UV–Vis spectroscopy (Perkin Elmer lambda 35 (Waltham, MA, USA)), FT-IR spectroscopy (Perkin Elmer 1000 FT-IR spectrometer) and XRD (D_2_ Phaser X-ray diffractometer (Bruker, Germany), Cu Ka radiation (k =1.5418 A °). HRTEM analysis is carried out using high resolution transmission electron microscopy (HRTEM) supplied by JEOL Model JEM 2100F operating at 200 kV. The surface morphology of the nanocomposites was determined by scanning electron microscopy (SEM, JEOL, JED-2200 series, Tokyo, Japan).
